# Reevaluating the safety of chamomile poultices in ophthalmic care

**DOI:** 10.3389/fphar.2025.1580586

**Published:** 2025-05-12

**Authors:** Tibor Rák, Adrienne Csutak

**Affiliations:** ^1^ Department of Pharmacognosy, Faculty of Pharmacy, University of Pécs, Pécs, Hungary; ^2^ Department of Ophthalmology, Medical School - Clinical Centre, University of Pécs, Pécs, Hungary

**Keywords:** chamomile, Matricaria recutita, ocular poultice, conjunctivitis, hypersensitivity, herbal tea

## Abstract

**Introduction:**

German chamomile [*Matricaria recutita* L. (Asteraceae)] tea poultices are a common folk remedy in Central and Eastern Europe for treating eye inflammations such as blepharitis and conjunctivitis. This practice often precedes medical consultation and professional advice. Surprisingly, some healthcare professionals, including pharmacists, naturopaths, general practitioners, and pediatricians, recommend it as a primary treatment, particularly among the elderly. However, the ophthalmic use of chamomile lacks scientific validation, and the European Medicines Agency (EMA) and ophthalmic guidelines warn of potential allergic reactions, including life-threatening anaphylaxis.

**Methods:**

This review examines the origins and descriptions of herbal poultices, with a focus on chamomile, by analyzing data from Hungarian and international medical literature. The study highlights the unsanitary production and storage conditions, also the external application of chamomile teas, which increase the risk of infection and contamination.

**Results:**

The findings indicate that chamomile tea poultices pose significant health risks due to potential allergic reactions and microbiological contamination. The study underscores the lack of scientific evidence supporting the ophthalmic use of chamomile and the dangers associated with its application.

**Discussion:**

The implications of these findings are critical for healthcare professionals and guideline developers. The study calls for discontinuing chamomile-based ophthalmic therapies and emphasizes the need for evidence-based practices. Future research should focus on validating the safety and efficacy of herbal remedies and developing guidelines to protect public health.

## 1 Introduction

Traditional folk medicine is defined as health practices, knowledge, and beliefs based on plant, animal, and mineral substances, spiritual practices, and manual techniques for disease treatment, diagnosis, and prevention, as well as the maintenance of overall wellbeing (Al-Ghadeer and Al-Amry, 2021; World Health Organization, 2002). Traditional and evidence-based medicine recognizes 50–70,000 of the world’s 250,000 flowering plants (Jarić et al., 2018; World Health Organization, 2019). Phytochemical analyses were performed on 15% of the currently identified plant species, with biological activity investigated for only 6% (Jarić et al., 2018). In developing countries, 48.7%–65.7% of patients reported consulting a traditional folk healer to address issues with their eyes (Atawi et al., 2024), yet the use of unprofessionally recommended natural substances by these healers contributes to 8%–10% of corneal blindness (Al-Ghadeer and Al-Amry, 2021). In some regions of the world, e.g., in India the most popular therapies for conjunctivitis and tear duct obstruction (Srivastava et al., 2010) are poultices made of grated potato, cucumber, black tea, or onion (Al-Ghadeer and Al-Amry, 2021; Atawi et al., 2024) applied to swollen eyes, and these practices are also exist in Hungary. Between the 18th and 20th century, Hungarian folk eye treatments have utilized a variety of ingredients and tools, such as breast milk, sour milk, powdered sugar, powdered glass, honey, urine, feces, chamomile, and more without success (Vámos, 2024). Tea-based eye poultices containing *Matricaria recutita* L. (Asteraceae), *Euphrasia rostkoviana* Hayne (Orobanchaceae), and *Juglans regia* L. (Juglandaceae) leaf are the current modern popular “remedies” in Hungary, applied by naturopaths, family doctors, and even pediatrician colleagues for conjunctivitis treatment without any official recommendation. Unfortunately, there is no exact data available to-day on the rate of ocular home remedies applied by Hungarian patients. According to Saudi Arabian statistics, the most popular remedies for conjunctivitis are *M. recutita* in 19.6% of the cases (Al-Ghadeer and Al-Amry, 2021). Given that *M. recutita* is widely recognized by nearly every Hungarian for its use in treating eye inflammation (Makay and Kiss, 1988), even beyond the borders, such as in Transylvania (Romania). In certain villages, chamomile ranked first in ethnopharmacobotanical interviews among the locals (Erdei et al., 2011). Based on these Transylvanian folk surveys, it was also revealed that there is an overlap in the use of herbal remedies, such as chamomile poultices, with the famous “*Maria Treben*” book in Central and Eastern Europe (Erdei et al., 2011). This publication is frequently referenced both in folk laymen and by patients visiting eye clinics. Based on the survey conducted by the Hungarian Community Agricultural Marketing Centre, chamomile teas were one of the most frequently (40%!) purchased herbal items (Erdész and Kozak, 2008). Currently, there is limited specific survey data on the use of *M. recutita* eye poultices among Hungarians or Eastern Europeans. Detailed epidemiological data on its application for eye health is scarce, affecting the ability to provide evidence-based recommendations and understand the risks. Chamomile eye poultices, rooted in traditional and folk medicine, are not systematically documented in modern surveys. Most research focuses on chamomile’s general medicinal properties, leaving a gap in data on its specific use for eye health. The scientific knowledge and data collected by the authors in this study can provide a basis for a more rigorous review of folk medicine practices, such as chamomile eye poultices.

In this study, the authors aim to explore the ethnopharmacobotanical and cultural origins of chamomile eye poultices used for treating eye inflammation. Additionally, they will examine its status and psychosociological perception among Eastern European patients. The study illustrates the potential dangers of chamomile poultices, contributing to the development of safer medical practices. The findings of this research can help healthcare professionals better inform patients about the benefits and risks of alternative treatments, thereby supporting informed decision-making.

## 2 Assessment of clinical problem and its implications

### 2.1 Chamomile in historical and ophthalmological perspective in Hungarian medical literature

The authors evaluated the *Arcanum* digital archive of Hungarian scientific literature for the keywords “chamomile” or “Matricaria” from issues of the *Orvosi Hetilap* and *Szemészet* (*Ophthalmologia Hungarica*). The following section presents our results in chronological order (Table 1). The involved literature data clearly demonstrates the broad medical usage of chamomile poultices for ophthalmic and other general disorders dating back 167 years; nevertheless, there is no mention of side effects or complications. It is important to acknowledge the limitations of using historical, non-peer-reviewed sources and the absence of adverse outcome data, which may affect the comprehensiveness and reliability of the findings. Although applying chamomile to the eyes appears to be a unique Hungarian practice, it should be noted that this folk medicine tradition of applying chamomile poultices to ocular inflammation exists in some parts of Spain (Granada, Catalonia), Italy (Campania), Eastern Serbia (Rtanj highlands), and Palestine. This practice is reminiscent of Greco-Roman medicine and its successor, the historical and geographic spread of Unani medicine (Mihyaoui et al., 2022). It was commonly regarded as a panacea in both European and Unani medicine (Sah et al., 2022). External eye problems, such as conjunctivitis (called “*Ashob-i-Chasm*” in Unani medical terminology) and obstruction of the lacrimal ducts, were also treated by this herb (Aaqil et al., 2022).

**TABLE 1:** Self-edited table summarizing the results of occurrence of chamomile in Hungarian medical literature.

Year	Author	Case/Description	Treatment/Application	Note	Reference^a^
1857	János Barlay	Hungary’s second case of cysticercosis: a 24-year-old woman with a lesion in her left eye	Chamomile tea poultice	Etiology: The patient contacted a child infected with tapeworms	Barlay (1904)
1872	Ferenc Torday	Therapy for keratitis pustulosa	Compressing bandage, chamomile poultice, atropine eyedrop, chlorine water (aqua chlori), application of silver nitrate, saline water	In severe cases, Graefe-Saemisch iridectomy is necessary	Torday (1872)
1879	Zsigmond Vidor	Case report of kerato-iritis cum hypopyo	Lifestyle modification, warm chamomile tea poultice, atropine eyedrops, potassium iodide ointment, mercury application (2 g)	The patient recovered	Vidor (1879)
1905	Vilmos Leitner	Purulent conjunctivitis in neonates and infants	3% boric acid, chamomile eye wash, removal of purulent discharge in every 4 h	The author emphasizes the frequent eye washing of the neonates by their mothers	Leitner (1905)
1910	Dezső Békés	A 17-year-old female worker with febrile TBC-related left eyelid edema and diplopia	Chamomile poultice, oral aspirin	Improvement by the third day	Békés (1910)
1930	Miklós Ács	Fluor vaginalis treated with chamomile tea vaginal rinses	Chamomile tea vaginal rinses	Has an astringent effect	Ács (1930)
1930	Vilmos Leitner	Treatment of tuberculous uveitis with tuberculin	Tuberculin injections, chamomile poultice, atropine eyedrops	Regression of Koeppe and Busacca nodules	Leitner (1903)
1938	Péter Jost	Conjunctivitis and inflammatory eye diseases	In case of external injury and rubbing of the eyes, chamomile eye poultice	In case of unsuccessful treatment, lifestyle adjustment is recommended	Jost (2022)
1943	Pál Ujsághy	Enteral toxicosis in infants	Chamomile tea with activated charcoal for stomach and bowel rinses	Effective against dehydration	Ujsághy (1943)
1948	Aladár Kettesy	Eyelid edema and eyelash misalignment in a 32-month-old infant	Surgical eyelid correction (Chamomile poultice was ineffective)	Chamomile may have exacerbated the edema, but it is a retrospective speculation	Balázs et al. (1962)
1957	Pál Weinstein	Introduction of Chame^™^ eye ointment to Hungarian medical readers	Eye ointment containing chamomile extract	A rarity among products marketed in Germany	Weinstein (1957)
2020	Ildikó Süveges	Textbook recommendation for Meibomian gland dysfunction and blepharitis	Chamomile eye wash or poultice followed by antibiotic ointment	Recommended in a general ophthalmology textbook for medical students	Süveges (2020)

^a^ The sources are detailed in references.

### 2.2 The survival of folk traditions–The “maria Treben phenomenon”

In this chapter, the authors address the critical ophthalmological and psychosociological evaluation of the widely known, non-scientific, and often harmful information contained in the “*Maria Treben*” book, which is popular in Central and Eastern Europe.

*Maria Treben* (1907–1991), an Austrian writer born under the Austro-Hungarian Empire, became a traditional “herbalist” after using herbs to treat terrible diseases in her family (Kerckhoff, 2020). She summarized her own therapies and published her main work, “*Health Through God’s Pharmacy*,” in 1980, at the age of over seventy, which has been published in over 80 editions and translated into 27 languages. The authors quoted from *Maria Treben*’s book is notable for the remedies indicated for eye treatment, allowing us to comprehend our patients’ naive therapeutic choices: in the case of *M. recutita*, “*externally … is used as a compress and a wash for inflamed eyes, conjunctivitis*” (Treben, 2023). She additionally referenced the Swiss herbalist Abbé Johann Künzle’s method: “*against eye-pain Camomile boiled in milk was applied as a compress over the closed eyes which healed in a short time*” (Treben, 2023). “*Weak eyes are strengthened if the freshly pressed juice of the Calamus roots* (*Acorus calamus* L. (Acoraceae)) *is brushed over the closed eyelids from time to time, the juice being left on the lids for a few minutes and rinsed off with cold water*” (Treben, 2023). *A. calamus* is on the Hungarian National Center for Public Health and Pharmacy’s prohibited list because of its hepatocellular carcinogenic component (asarone) (OGYÉI, 2023). The book promises that if the eyes are cleansed with warm *Calendula officinalis* L. (Asteraceae) tea, as well as the effect of a sitz bath with *Equisetum arvense* L. (Equisetaceae) on the kidneys, the patient’s “visual impairment would be eliminated” (Treben, 2023). *Taraxacum officinale* L. (Asteraceae) is also cited as an eyewash ingredient (“*they used to wash their eyes with it*”) (Treben, 2023), but the authors did not discover any references to this in the academic research on phytotherapy outside of the book. The skin-corrosive *Chelidonium majus* L. (Papaveraceae) is a good example (Maji and Banerji, 2015) of how the book’s description can also promise consequences that jeopardize vision. The book’s presentation is not only unscientific, but it nearly contradicts ophthalmological facts: „C*ataract and spots on the cornea are caused to disappear gradually. The juice even helps in cases of a bleeding or detached retina. A leaf of the Celandine is washed and the stem of the leaf is rubbed between the wet thumb and index finger. The juice thus won is brushed gently over the closed eyes towards the corners. Although not rubbed into the eyes, they nevertheless benefit from it. This holds good for cataract and defective vision and is prophylactic for healthy but strained eyes*” (Treben, 2023). The issue is that Maria Treben, knowing neither ophthalmology nor phytotherapy, exposed a patient at risk with her statement that „ *I told her that it was harmless to the eye*” (Treben, 2023). The patient’s eyelid was treated with the orange sap of the herb, and it was later recognized as a malignant tumor based on contemporary descriptions. Although the book neglects to mention, it was most likely a basalioma of the eyelid. The fresh yellowish milky sap of *Ch. majus* can stain the skin when applied directly, and its slightly acidic (pH 4) chemical impact produces irritation, photosensitivity, and in some cases, allergic contact dermatitis with skin blistering (Maji and Banerji, 2015). The authors strongly discourage its usage on ocular tissues! Maria Treben treats cataract and glaucoma uniformly, which is neither pharmaceutically nor professionally correct. For cataracts, she advises Bitter Swedish Drops compresses, and for glaucoma, a 20-min sitz bath made from *E. arvense* “*since they relieve the pressure from the eyes*,” which involves soaking overnight and cooking the next day. From a professional standpoint, such a delay is inappropriate in a patient with acute angle closure or suboptimal intraocular pressure. She recommends “*eye steaming*” with a professionally uninterpretable herbal mixture diluted in white wine, and if the patient has unpleasant eye symptoms, she recommends treating them with a Bitter Swedish Drops compress (Treben, 2023). She also created a similarly unintelligible herbal concoction for lacrimation (“*weeping*”), which contains 10 g *Valeriana officinalis* L. (Caprifoliaceae), 10 g *Cnicus benedictus L.* (Asteraceae), 10 g *Syringa vulgaris L.* (Oleaceae), 15 g *Alchemilla vulgaris L.* (Rosaceae), 10 g *Ruta graveolens L.* (Rutaceae) (Treben, 2023). According to its description, “*eyes can be bathed*” by *E. rostkoviana* “*but the infusion has to be very weak*” (Treben, 2023). The text does not specify how “weak” the solution should be (“*only half a teaspoon*”) or what the maximum permitted dose is. She asserts: “*if the trouble worsens after this treatment, the infusion was too strong*” (Treben, 2023). In practice, the reader is liable for any ocular issues that ensue. It is favorable because it allows the freshly made infusion to be utilized only once (Treben, 2023).

The most serious issue with the book and Maria Treben’s theories is that the majority of the content is her own hypothesis, which is not scientifically validated and does not correspond to any internationally acknowledged phytotherapy recommendations. The author is not a professional, but rather a lay folk writer, and she can be considered a reputable source for anyone looking for similarly lay folk “simple” remedies. Patients attending for outpatient and emergency ophthalmology care at the University of Pécs Department of Ophthalmology applied homemade chamomile tea poultices and eye-washes to treat their conjunctivitis of various etiologies and clinical stages. The similar customs could be observed in other Hungarian ophthalmology wards. Because of an upsurge in allergies and other inflammatory reactions caused by multiple-day treatment, they can occur even in late-night ophthalmology emergency care. The authors have included these cases as illustrative examples to provide valuable insights and lessons for the readers (Table 2). During the history taking, the patients stated that her own therapeutic beliefs based on the „*Maria Treben* book”. Especially *Case 3* ultimately prompted the authors to investigate the background and implications of these folk practices.

**TABLE 2:** Self-edited table summarizing some recent examples of *M. recutita* poultices and their adverse events among patients visited University of Pécs Clinical Centre Department of Ophthalmology.

Case	Year	Patient details	Initial treatment	Complications	Outcome
1	2020	84-year-old female, injured by grandchild’s toy car	Home-brewed *M. recutita* tea poultice	Irritation in the second eye, delayed clinic visit	Surgical management of perforated cornea with prolapsed iris
2	2023	40-year-old female, inflamed eye	Brewed *M. recutita* tea poultice (left in kitchen for 5 days)	Mucopurulent preseptal cellulitis, facial erythema	Emergency room treatment
3	2023	Female with elevated intraocular pressure (48 mmHg) due to acute angle closure glaucoma	Bitter Swedish Drops (Nagy Svédcsepp^™^, Naturland, Hungary; containing 40V/V% alcohol!) concentrate, *Achillea millefolium* L. tea poultice, vasoconstrictor (tetryzoline hydrochloride) eye drops	Ineffective home treatment, referred to ophthalmology outpatient unit	Investigation into folk practices prompted
4	2025	54-year-old male, postoperative cataract surgery control	Dried yellow discharge from the operated eye surface and eyelids was washed with *M. recutita* tea	Eyelid swelling and severe inflammation of the operated eye	Discontinuation of practice, patient education, topical steroid and antibiotics

According to the examples above, traditional folk medicine beliefs persist in Eastern Europe, especially in Hungary, therefore the chamomile tea eye poultice takes precedence over medical examination and professional counsel (Fazekas, 2010). In her mini-report, *Piroska Fazekas* emphasizes the importance of seeking professional medical advice promptly, because certain home remedies found on the internet (in Hungary), such as chamomile poultices, may exacerbate certain (eye) conditions if misdiagnosed and improperly treated at home (Fazekas, 2010). The “axiom” that has become ingrained in nations’ collective consciousness is based on the principle of similarity found between traditional folk medicine elements, i.e., analogical thinking (Györfi, 2016) states that “*the chamomile flower resembles the eye, therefore it is good for the eye*”. The survival of the method is further supported by the fact that scientific medications are frequently expensive, as evidenced by the cost of antibiotic-containing eye drops or the worry of the possible adverse effects (Györfi, 2016). Furthermore, overcrowding of ophthalmology outpatient departments, fear of medical professionals, and barriers to receiving ophthalmology care may compel patients to employ potentially dangerous traditional remedies (Györfi, 2016). Demand for natural remedies, as well as religious confidence in “ancient European” treatments (Zörgő et al., 2016), contribute to the popularity of *M. recutita* tea poultices. Thus, if a competent ophthalmologist or naturopath recommended a patient against using chamomile poultices, the providers would be accused of being manipulated by the “Big Pharma lobby” (Zörgő et al., 2016). “Pharmaceutical mafia” conspiracy theories and “nothing synthetic” perspectives support unproven “natural” therapies (Zörgő et al., 2016), ignoring the fact that naturopathic product distribution is sometimes ultimately a marketing and business issue. The refusal of synthetic pharmaceuticals may be primarily motivated by broader society values, despite the fact that herbal extracts constitute basically the raw materials of our modern medications and may be supported by scientific studies and evidence (Rák and Csutak, 2024a; Zörgő et al., 2016). Economic background is another interesting approach: for herbs, and supplements, according to the surveyed Hungarian informants, the primary consideration in purchasing is the price of the raw materials. Currently, the ingredients of traditional Hungarian products, such as *M. recutita*, mostly come from imports e.g., Egypt (Erdész and Kozak, 2008). Erdész and Kozak noted in their report that the primary reason for relying on imported herbs is their lower cost compared to Hungarian raw materials. Uncritical “experiences” of patients, referencing to our “grandmothers” who have experienced everything, or the healing of others, rendering comprehension more difficult (Zörgő et al., 2016). “*The basis for decision-making shifted from institutions to individuals, and experience increasingly became the source of authenticity, rather than tradition*” to quote from article by *Szilvia Zörgő*, which describes the consequences of the enormous information explosion of our modern scientific-technological civilization. The transmission of personal, sometimes non-professional, but highly subjective religious experiences and preferences plays an important part in therapeutic selection (Zörgő et al., 2016). Among laypeople, respected traditional healers have risen to the position of “experts” in Eastern Europe (such as in Hungary). Their historical expertise and wisdom deserve to be respected, yet some of their practices are not ophthalmologically or phytotherapeutically appropriate.

### 2.3 Effects on the ocular surface

The epithelial coating of the eye surface serves as a barrier, meaning it is constantly interacting with the external environment and actively removes foreign particles. The reflex blinking of eyelid offers physical and chemical support for the tear-film build-up as well as a shifting resident microbiota (Barcsay-Veres, 2022). Conjunctival-related lymphoid tissue (CALT) and lacrimal drainage-related lymphoid tissue (LDALT) contribute to preserving the ocular surface immunotolerance (de Paiva et al., 2022). Antigenic tolerance is a consequence of an active process in which the conjunctiva’s goblet cells play a crucial role. They deliver a “tolerance signal” to the dendritic cells of the conjunctival stroma, preventing particular immune activation and inflammation (Barcsay-Veres, 2022). The molecular pattern recognition elements of the innate immune response typically identify and effectively eliminate antigens, but the adaptive immune system is additionally activated on the ocular surface through the involvement of antigen-presenting cells (APCs). The lymph nodes activate circulating naive T cells, and then, with the support of B cells, specific antibodies are transported to the lacrimal gland and the eye surface via the bloodstream (Barcsay-Veres, 2022). Coordinated neuro-immuno-hormonal mechanisms that can be linked to the ocular surface enable active immune protection and tolerance, both of which are required for the ocular surface to be functioning properly (Barcsay-Veres, 2022). Damage to the protective proteins of the lubricating tear film, including vitamins, IgA, TGF-β, retinoic acid, lysozyme, lactoferrin, and trefoil peptide, as well as the number or function of conjunctival goblet cells, can compromise the homeostasis and immune protection of the ocular surface (Barcsay-Veres, 2022; de Paiva et al., 2022). During inflammation, plasma proteins (such as albumin) are produced from the lacrimal gland and conjunctival vessels, binding and inactivating several protective proteins (Barcsay-Veres, 2022). The production of matrix metalloproteinases (e.g., MMP-9) and cytokines (e.g., IL-33), which alter tight junction protein networks, contributes to the breakdown of the corneal epithelial barrier in autoimmune conditions such as allergic conjunctivitis and dry eye disease (de Paiva et al., 2022).

**FIGURE 1:**
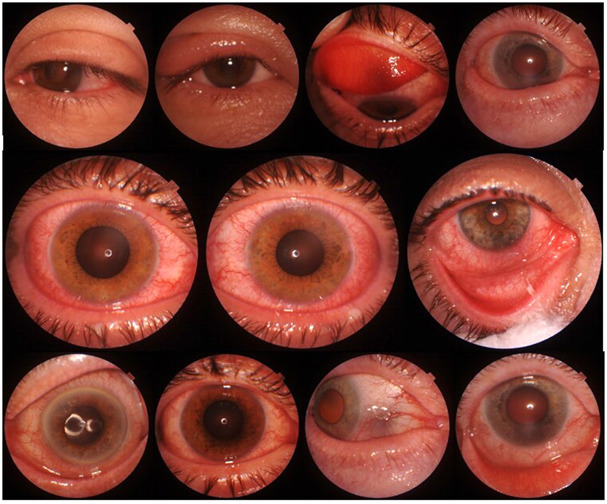
Optical Coherence Tomography (OCT) device with Triton DRI OCT fundus camera function (Topcon Healthcare, Japan) showing montage of ocular sequelae after chamomile tea poultice (mixed conjunctival injection, blepharitis, eyelid edema, conjunctival chemosis). The applied Matricaria recutita monoherbal tea products were presented to the treating physician to identify the causing agent.

Type I hypersensitivity is responsible for the allergic symptoms of chamomile eye poultice (Módis and Süveges, 2023; Reider et al., 2000). When allergenic pollen contacts the eye’s surface, they cause an inflammatory reaction, resulting in conjunctivitis. At the primary contact, no irritation develops, leading the unprepared patient to believe that the chamomile poultice is innocuous; however, the creation of allergen-specific IgE immunoglobulins begins. IgE sensitizes mast and basophil cells, allowing them to release proinflammatory cytokines and prostaglandins. It also activates eosinophil cells, which create a variety of interleukins, so commencing and accelerating the inflammatory process during the following week (Módis and Süveges, 2023; Reider et al., 2000). An acute reaction occurs promptly or within minutes of being exposed to the recognized allergen. Sensitized cells release a high amount of histamine, which once it reaches the target organs causes inflammatory symptoms (Módis and Süveges, 2023; Reider et al., 2000). As a continuation of an acute reaction, in the case of symptoms that appear after a few hours or days, a late-type reaction occurs (Módis and Süveges, 2023; Reider et al., 2000), in which case the affected patients cannot immediately consider contact with chamomile as an etiological factor. Experiencing the “severity” of the eye disease, they search for an ophthalmologist after several unsuccessful applications of poultice. The authors are unaware of chronic relapsing chamomile exposure inflammation that lasts days, months, or years. IgE hypersensitivity and mast cell activation increase the level of histamine, tryptase, and prostaglandins in the tear film (Módis and Süveges, 2023; Reider et al., 2000). Vascular endothelial cells, along with Th-1 and Th-2 lymphocytes, are activated, promoting the release of different inflammatory factors. These contribute to patients’ subjective symptoms, such as tearing, burning sensations, conjunctival vascular dilatation, and chemosis. Sesquiterpene lactones present in chamomile eye washes and poultices develop contact dermatitis (late type IV allergic reaction), which mostly affects the eyelids and the periorbital skin, resulting in eczematous hyperaemic inflammation and edema (Módis and Süveges, 2023; Reider et al., 2000). The aforementioned ophthalmic symptoms and signs were also observed in patients at the University of Pécs Clinical Centre Department of Ophthalmology (Figure 1), causing them severe ocular discomfort. Pollen antigens are low-molecular-weight substances that interact with cutaneous proteins. A similar reaction occurs in case of chronic application of antiglaucoma eye drops as adverse effects (Módis and Süveges, 2023).

### 2.4 The official statement of the European medicines agency

Traditional European folk medicine applications of *M. recutita* involve bath additives with liquid extracts (tea, essential oil, tincture), as well as adjuvant therapy for skin irritation, particularly in the anogenital region (EMA, 2015; Szabó, 2016). The active components work by blocking the cyclooxygenase (COX) enzyme. When used topically as ointments or poultices, it lowers inflammation of the skin and mucous membranes while also promoting the healing of surface lesions. It has also been shown to be effective in treating inflammations of the oral and stomach mucosa. Its effects have also been confirmed in human studies. Internally (e.g., as tea), it can be used to treat ulcers and gastritis because it lowers gastric juice secretion and gastrointestinal mucosal inflammation (EMA, 2015). On a daily basis, the global consumption of chamomile tea exceeds one million cups (Guzelmeric et al., 2017).

The monograph’s principal contraindication is hypersensitivity to the active components in *M. recutita* and other Asteraceae species (EMA, 2015). Chamomile allergy can be classified as part of pollen-food syndrome, commonly known as *mugwort-birch-celery-spice syndrome* (Reider et al., 2000). Cross-reaction generated by food and plant pollen in Artemisia spp., and other plants belonging to the Asteraceae family (about 20,000 recognized species) is known as IgE-mediated type I hypersensitivity (Andres et al., 2009; Reider et al., 2000). Seasonal allergic rhinoconjunctivitis, atopic eczema, and plant pollen allergy are all known in high-risk patients (Andres et al., 2009). To understand the adverse effects of M. recutita, the authors have briefly summarized the current knowledge on the potential molecular agents responsible. The characteristic apple-like scent of chamomile [Ancient Greek term for “earth apple,” χαμαίμηλοv (chamēmilon)] is primarily attributed to the presence of certain volatile compounds e.g., esters such as butyl angelate, isobutyl angelate and isoamyl angelate, and terpenoids like α-bisabolol, and chamazulene (Parveen et al., 2023). The chemical composition of the plant is impacted by the geographical distribution and genetic variability of the species. *Wesolowska et al* reported α-bisabolol and α-bisabolol oxide A as predominant chemotype (23.9%–44.2%) in the samples from Moldova, Russia, Germany, Hungary and Czech Republic (Mihyaoui et al., 2022; Wesolowska et al., 2015). The sample from Armenia was rich in α-bisabolol oxide B (27.2%) and chamazulene (15.3%) (Wesolowska et al., 2015). *Mavandi et al* identified three chemotypes in the essential oil of *M. recutita* from Iran (α-bisabolol oxide A, α-bisabolol oxide B, and α-bisabolon oxide) (Mihyaoui et al., 2022). In Portugal and Spain, *M. recutita* samples contain higher α-bisabolol content, while in Bulgaria and Turkey the oxidative forms, like α-bisabololoxide A and α-bisabololonoxide A are more prevalent (Wilkinson et al., 1995). In Argentina, α-bisabololoxide B rate was higher than the European samples (Wilkinson et al., 1995). Bisabolol (a sesquiterpene alcohol) is oxidized to α-bisabololoxide A, B and C, and α-bisabololonoxide A (Wilkinson et al., 1995), which oxidative forms showed moderate senzitizing potential in murine models (Wilkinson et al., 1995). Hydrophilic components are mostly terpenoids (Navarro-Triviño et al., 2022). Main components of aqueous tea extract are polyphenols (e.g., apigenin, luteolin, quercetin, patuletin), but contains matricin and desacetyl matricarin (both sesquiterpene lactones), and herniarin (7-methoxycoumarin), which are described as potential allergens causing chamomile-related dermatitis (Navarro-Triviño et al., 2022), however all potential allergens are not fully elucidated yet. Wilkinson et al. also mention, that desacetyl matricarin produced positive patch reaction on their patient (Wilkinson et al., 1995). *Ramazani et al.* reported α-bisabolol containing up to 0.5% caused skin irritation in guinea pigs and humans (Ramazani et al., 2022). *Paulsen et al.* investigated *M. recutita* sensitivity (Hungarian chamomile was also tested) on human subjects. 8 of the remaining 12 patients had 1 or more positive reactions to chamomile-containing preparations: 3 positive test to chamomile tea, 6 to chamomile creams/ointments and 1 to chamomile oil (Paulsen et al., 2008). According to their results, the overall prevalence of positive reactions to chamomile- (and/or arnica-)containing preparations was 72% (Paulsen et al., 2008). All patients were positive to the oleoresinous chamomile extract, and among the 11 commercial stay-on products containing *M. recutita*, 7 elicited positive reactions (Paulsen et al., 2008). *Travassos & Goossens* also observed sensitizations to bisabolol in some human cases (Travassos and Goossens, 2012). However, the Cosmetic Ingredient Review Expert Panel noted that since bisabolol enhances the skin penetration of other ingredients, cosmetic manufacturers should exercise caution when combining bisabolol with ingredients that could be toxic if absorbed by the skin (Alan Andersen, 1999). Overall, the Expert Panel concluded that bisabolol was safe as used in cosmetics and personal care products (Alan Andersen, 1999). Despite information regarding allergies, the *United States Food and Drug Administration* (FDA) also considers α-bisabolol safe due to its low toxicity and has classified it as Generally Recognized as Safe (GRAS) (Ramazani et al., 2022).

Exact incidence and prevalence data on allergic reactions and anaphylaxis cases are unavailable from research or the EMA and ESCOP monographs (EMA, 2015; ESCOP, 2020; Reider et al., 2000). Severe allergic reactions (dyspnea, Quincke’s edema, vascular collapse, and life-threatening anaphylactic shock) have already been recorded when chamomile products are administered to the mucosal membrane (EMA, 2015). Although sensitization has been described with a variety of chamomile preparations, including chamomile oil, chamomile tea, and sesquiterpene lactone-free chamomile cultivars, too (Paulsen et al., 2008). *Florido-Lopez et al.* discourage the readers against ocular use of chamomile, according to their results first in the literature applying Chamomile Conjunctival Provocation Test with Prick’s test (Florido-Lopez et al., 1995). All their patients (n = 20) presented clear positive conjunctival reactions to *M. recutita* aqueous extract even at low level (1/10 and 1/100) dilutions (Florido-Lopez et al., 1995). *Subiza et al.* reported that seven patients who cleansed their eyes with chamomile tea suffered severe allergic conjunctivitis, with two patients also developing angioedema (Fraunfelder, 2008). *Andersen et al.* reports that undiluted α-bisabolol was instilled into conjunctival sac of rabbits without rinsing the eye (Draize test). No corneal adverse effect was observed; however, well-defined conjunctival redness was noted in all rabbits at the 1, 24, and 48 h. Increased discharge was noted in all animals at the 1 h reading (Alan Andersen, 1999). *Wilkinson et al.* reported a 37-year-old woman with periorbital, facial and forehead edema after application of chamomile-containing cosmetics (Wilkinson et al., 1995). An allergic reaction was previously described in the case of a chamomile enema given in a laboring female patient, resulting in fatal fetal hypoxia in the unborn child. A preoperative chamomile enema administered to another female patient provoked bronchospasm, colic symptoms, vomiting, and loss of consciousness within 5 min (Reider et al., 2000). *Reider et al.* observed of chamomile-induced anaphylactoid systemic symptoms in ambulatory patients, including rhinoconjunctivitis, stridor, dyspnea, vomiting-diarrhea, urticaria, angioedema, and hypovolemic shock (Reider et al., 2000). It is noteworthy that chamomile is also mentioned as an occupational hazard, with rhinoconjunctivitis and asthma developing as a result of allergy-related sensitization in workers at a tea filter manufacturer (Vandenplas et al., 2008). Clinicians advise patients against using chamomile poultices due to cross-reactivity with allergens found in *M. recutita* pollen and other allergenic pollens, as well as established atopic illnesses (Fraunfelder, 2008). Finally, poultices produce eyelid edema, conjunctival chemosis, and allergic hyperemia, which conceal the symptoms of the underlying eye condition, therefore treating inflammatory reactions is also required, making it difficult to adequate patient care (Subiza et al., 1989). These findings are supported by a summary of the ocular adverse effects of medicinal plants published in the *American Journal of Ophthalmology* and by *Santaella* and *Fraunfelder*, which was classified as having high evidence (Fraunfelder, 2004; Santaella and Fraunfelder, 2007). There is no special recommendation for this case in the literature, but if the patient has a known allergic reaction with anaphylaxis, it is recommended that the patient carry a portable epinephrine injection (e.g., Anapen^TM^, Bioprojet Pharma, France) because anaphylaxis-induced hypovolemic shock, vascular collapse, and Quincke’s edema are life-threatening complications (Andres et al., 2009). Intravenous antihistamines and corticosteroids have been shown to ameliorate chamomile-induced anaphylactic responses in emergency conditions (Andres et al., 2009; Reider et al., 2000).

In the majority of the literature on chamomiles in this article, it is declared that the species in question is *Matricaria recutita* (synonym *Matricaria chamomilla*). However, it cannot be excluded that studies referring only to “chamomile” might be discussing a different, similar species. Our clinical data identifies the species used in tea preparations as *M. recutita*, which has been confirmed by laboratory analyses conducted by the University of Pécs Faculty of Pharmacy. These analyses support that the ocular complications in various patients were caused by an ocular poultice made from *M. recutita*.

### 2.5 Microbiological purity–Brewing is insufficient

**FIGURE 2:**
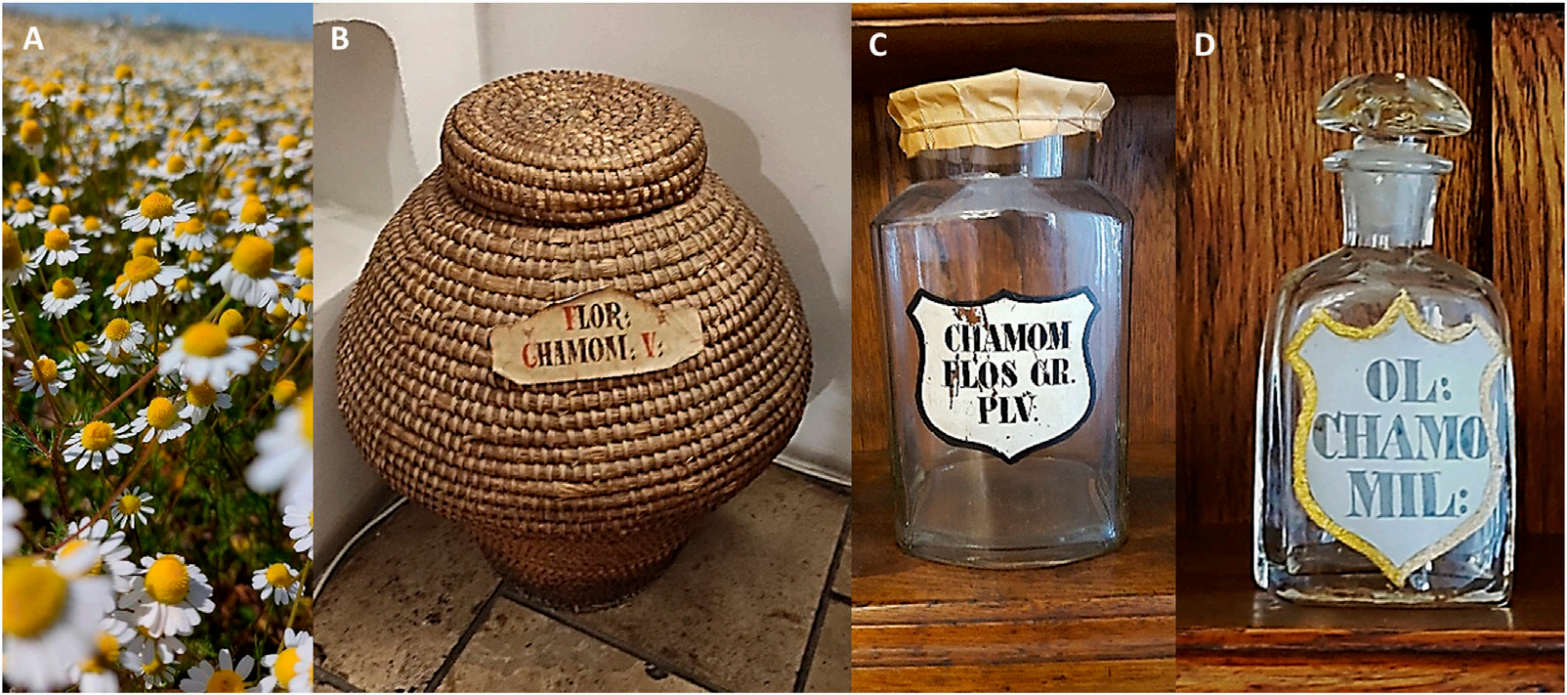
Chamomile flower (Matricariae flos - **(A)**; photographed by Zoltán Rapp, Püspökladány, Hungary, 2024). Apothecary storage possibilities of chamomile in wicker basket **(B)**; Golden Eagle Pharmaceutical Museum, Budapest, Hungary, 2023), or solid herbal drug **(C)**; Black Eagle Pharmaceutical Museum, Székesfehérvár, Hungary 2022) and essential oil **(D)**; Golden Lion Pharmacy, Kaposvár, Hungary 2019) containing vials (photographed by Tibor Rák).

*Matricaria recutita* is a common herb native to Southern and Eastern Europe (McKay and Blumberg, 2006; Singh et al., 2011). It is also grown in Germany, Hungary, France, Russia, the Balkans (McKay and Blumberg, 2006; Singh et al., 2011), and in remote places such as Brazil and Kenya is also cultivated mainly for export (Singh et al., 2011; Yamoah et al., 2014). The authors highlight this point because it can provide explanations for the contamination of imported chamomiles. Hungary is the leading producer of chamomile biomass (Singh et al., 2011; Sváb, 1979); it grows abundantly even on saline soil and is a main source of income for primary producers (Singh et al., 2011). Although it is also known as *Hungarian chamomile*, as it has been nominated as *Hungaricum* (a Latin term used to describe a unique value representing Hungarian culture and heritage) since 2015 (Singh et al., 2011; Szabó, 2016). This conclusion is additionally reinforced by the fact that, in 2016, *M. recutita* received the award for *Medicinal Plant of the Year* by the Hungarian Society of Pharmaceutical Sciences (Szabó, 2016) (Figure 2).

The vast majority of chamomile now originates from cultivation, which began in Hungary in 1958 and expanded to large-scale production in the 1980s. Aside from crop safety of produced chamomile cultivars, a significant research goal was to improve the quantity of beneficial active compounds (Szabó, 2016). The filtered teas include high-quality chamomile flour (*cribratum*), which must not contain foreign substances. In the 1930s, foreign materials, as 30 different plant species, were accidentally introduced to the mixtures, mostly from Russian imports (Szabó, 2016). Foreign plant materials, such as *Eremopyrum triticeum* (Gaertn.) Nevski (Poaceae), *Salsola tamariscina* Pall. (Amaranthaceae), *Matricaria discoidea*, and *Tripleurospermum inodorum*, are rarely found in the dried tea drugs under modern agricultural conditions, but are more common among hand-harvested process (Szabó, 2016). *Shimshoni et al.* reported that chamomile [(mean value of 261 µg/kg (range: 20–507 µg/kg)], peppermint and rooibos tea samples were the most heavily contaminated herbal teas with dehydro pyrrolizidine alkaloids in the Israeli market (Shimshoni et al., 2015). For example, drinking a beverage made from one teabag (assuming full transfer from the teabag to the water) containing 2 g of chamomile surpasses the recommended maximum tolerable daily intake of 0.49 µg dehydro pyrrolizidine alkaloids for a 70 kg adult and exceeds the recommended safe daily dose (0.04 µg) for infants weighing 5 kg by more than 13 times (Shimshoni et al., 2015). It is known that medicinal plants can absorb and store heavy metals from the environment (Kovács-Valasek et al., 2023; Rák and Csutak, 2024a). For example, *M. recutita* can accumulate considerable levels of cadmium in its roots (*radix*) and flowers (*anthodium*) (Singh et al., 2011). In the United Arab Emirates, *Dghaim et al.* found cadmium levels exceeding 0.82 mg/kg in 55% of chamomile samples (permissible maximum value: 0.3 mg/kg), and lead in 44 (5.37–11.40 mg/kg) (Özden and Özden, 2018). According to *Hrkić et al.* cadmium (0.053–0.248 mg/kg, lead (0.17–0.31 mg/kg) and chrome (0.32–0.66 mg/kg) can be detectable in smaller amount in Bosnian Herzegovinian chamomile samples, but iron (90.8–477.3 mg/kg) and copper (12.6–18.0 mg/kg) had higher content (Hrkić et al., 2021). This average values in herbal teas below the WHO’s permissible limits of 10mg/kg cadmium, 1.3 mg/kg lead, 0.3mg/kg chrome, and 30 mg/kg for copper while the limits of 450 mg/kg for iron (Hrkić et al., 2021).

In addition to foreign organic and inorganic substances, the microbiological purity of a given product is a very important public health issue. Chamomile poultice “enthusiasts” may argue that the herb itself has anti-inflammatory and antibacterial properties. Furthermore, brewing chamomile tea eliminates the microbes, thereby rendering it safeguarded for the eyes. In 2013, *Silva et al.* isolated antibiotic-resistant bacteria (*Staphylococcus aureus, coagulase-negative Staphylococcus, Pantoea agglomerans, Enterobacter cloacae*, and *Serratia ficaria*) from aqueous chamomile tea samples (Silva et al., 2013). Cold brewing teas increases potential hazards due to the lack of any thermal treatment. *Salmonella enteriditis* species can survive at 25°C in dried chamomile tea samples and at temperatures below 80°C (Keller et al., 2015). German chamomile samples had a substantially less bactericidal effect than ginger (*Zingiber officinale* Roscoe (Zingiberaceae)) and cinnamon (*Cinnamomum verum* J.Presl (Lauraceae)) (Liu et al., 2020). *Escherichia coli* exhibited the highest survival rate in chamomile teas (Liu et al., 2020). *Pantoea agglomerans* and other Gram-negative bacteria (e.g., *Bacillus* spp. and *Clostridium* spp.) were found in higher concentrations in Polish chamomile samples, which can tolerate harsh conditions (Esmi et al., 2022; Tournas and Katsoudas, 2008). In terms of microorganisms, fungal contamination (*Aspergillus* spp., *Eurotium chevalieri*, *Penicillium* spp., *Rhizopus* spp., *Rhodotorula glutinis*, and *Cryptococcu*s spp. in 50% of samples (Tournas and Katsoudas, 2008)) was also detected in chamomile teas, as opposed to *Jasminum officinale* L. (Oleaceae) teas, where the least was detected (Esmi et al., 2022; Tournas and Katsoudas, 2008). Higher levels of aflatoxin contamination have been documented in Portugal, Iran, and Spain, but in 2016, *Tosun et al* reported that 100% of chamomile tea consumed in Turkey was infected with Aflatoxin-B1 (Esmi et al., 2022; Özden and Özden, 2018). Detectable ochratoxin, DON toxin contamination is also not unknown, and according to *Esmi et al.*, ingestion of 3 cups of chamomile tea per day is sufficient to become a significant intake (Esmi et al., 2022). Iranian *M. recutita* tea samples can reach 40–100% Ochrtoxin-A mycotoxin, according to the study of *Esmi et al.* (Esmi et al., 2022). The University of Pécs Faculty of Pharmacy is additionally investigating fungal toxin contamination of chamomile teas, with the exact results expected to be released in the future. Although the majority of chamomile teas meet the criteria of the European institutions, 30% of chamomile teas include more molds than the limit set by e.g., Decree No. 461 of the Cabinet of Ministers of the Republic of Latvia (Reinholds et al., 2020). Improper industrial and home processing settings, as well as personal hygiene considerations, might be dangerous. Microorganisms and endotoxins promote eye infections and allergies (Tournas and Katsoudas, 2008). These bacterial and fungal species are known to cause a variety of eye illnesses, including endophthalmitis, which can potentially result in blindness and encephalitis.

**FIGURE 3:**
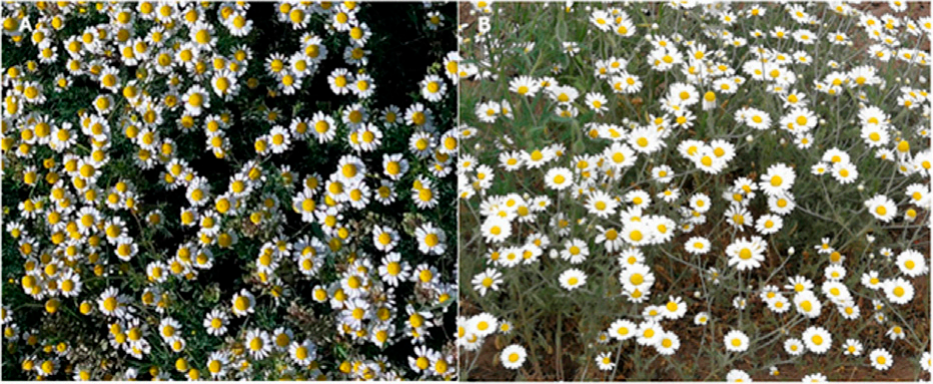
Matricaria recutita **(A)**; photographed by Zoltán Rapp, Püspökladány, Hungary, 2024) and Anthemis arvensis **(B)**; photographed by Erika Feller, Nyíregyháza, Hungary, 2022) in their natural place of occurrence. For laymen, they can be easily confused.

It is particularly problematic that the ancient descriptions refer to several similar plants under the name “chamomile” (in Unani medicine “*babuna*”): *Matricaria recutita*, *Chamaemelum nobile* (L.) All. (Asteraceae), *Corchorus depressus* (L.). Peterm. (Malvaceae), *Matricaria discoidea* DC. (Asteraceae), *Matricaria occidentalis* G. (Asteraceae), *Matricaria aurea* Loefl. (Asteraceae), *Anthemis arvensis* L. (Asteraceae), *Anthemis cotula* L. (Asteraceae), *Tripleurospermum inodorum* (L.) Sch.Bip. (Asteraceae) and *Cota tinctoria* (L.). J. Gay ex Guss. (Asteraceae), *Anthemis cretica* L. (Asteraceae), *Cota austriaca* L. (Asteraceae), *Anthemis coelopoda* Boiss. (Asteraceae), *Anthemis wiedemanniana* Fisch. & C.A.Mey. (Asteraceae), *Bellis perennis* L. (Asteraceae), *Glebionis coronaria* (L.) Cass. ex Spach. (Asteraceae) therefore, the plant descriptions in traditional medicinal literature are unfortunately inconsistent, resulting in an extensive amount of confusion, abuse, and drug counterfeit (Aaqil et al., 2022; Guzelmeric et al., 2017; Sah et al., 2022; Singh et al., 2011; Szabó, 2016) (Figure 3). *A. cotula* and *Senecio* spp. (Asteraceae) can be highly lethal (e.g., hepatotoxicity) so exchanging the herbs results in undesirable outcomes (García et al., 2020; Guzelmeric et al., 2017). After performing a thorough taxonomic and anatomical examination, *Ghauri et al.* concluded that the medicinal herb “babuna” relates to the species *M. recutita* (Aaqil et al., 2022; Sah et al., 2022; Singh et al., 2011). It is a severe concern, since *M. recutita* does not appear consistently even in Unani medicine and other ancient texts, and false identification of species can have serious implications.

The above may indicate reason for concern, especially if it is applied to a mucous membrane capable of absorption, such as the conjunctiva. The question also arises as to what sort of medicinal tea actually applied by the patient as poultice on the eyes, and what kind of hidden pollutant can be found in the package named “chamomile”. The warnings in official herbal monographs and case studies in the literature make it evident that recommending chamomile tea poultices for the eyes jeopardize our patients.

## 3 Actionable recommendations

Based on the scientific findings on the ocular risks associated with *Matricaria recutita* poultices (and other traditional herbal applications), the following recommendations should be considered for policy development and public health advisories:

### 3.1 Allergic reactions and immune response

Current regulations and recommendations vary widely, but the EMA monograph highlights *M. recutita* hypersensitivity, particularly in individuals with allergies to the Asteraceae family (EMA, 2015). Despite this, no strict regulations prohibit its use on the eyes, leaving patients vulnerable to type I hypersensitivity reactions (Andres et al., 2009; Reider et al., 2000). Given the well-documented allergic conjunctivitis and potential for anaphylaxis, stricter labeling and clinical warnings may be necessary.

### 3.2 Regulatory warnings and labeling

Explicit warnings must be mandated on commercial chamomile products, particularly herbal teas, stating that they must not be applied to the eyes. Contraindications for individuals with known pollen allergies and atopic conditions must be included in herbal product monographs. In Hungary, references to “folk medicinal knowledge” are used on the packaging of herbal teas to suggest ophthalmic applications. However, according to both *European Union Directive 2001/83/EC* and Hungarian regulations (*Decree No. 37/2004 (IV.26.) of the Minister of Health, Social Affairs and Family on food supplements* aligns with European Union regulations), health claims cannot be attributed to food products, unless they have been authorized as medicinal products. This includes the requirement that any health claims must be substantiated by scientific evidence and approved by e.g., the EMA or FDA. Based on the above data, the authors would extend the detailed discussion and expansion of ophthalmic contraindications in the official monographs of chamomile by EMA, *European Scientific Cooperative on Phytotherapy* (ESCOP), and other authorities.

### 3.3 Sterility and quality control standards

In Hungary, the relevant regulation is *Decree No. 37/2004 (IV.26.)* specifies that food products, including herbal teas, do not need to undergo the same stringent efficacy and microbiological contamination testing required for pharmaceuticals. At the European level, *Directive 2001/83/EC* outlines the requirements for medicinal products, including herbal medicinal products. According to this directive, herbal preparations classified as food products do not need to meet the same rigorous standards as medicinal products. Implement stricter microbial contamination screening for herbal products marketed for topical or ophthalmic use. Sterilization protocols must be established for any approved ocular herbal remedies, ensuring pharmaceutical-grade purity.

### 3.4 Microbial contamination risks

Studies indicate that brewing chamomile tea does not eliminate antibiotic-resistant bacteria (Esmi et al., 2022; Silva et al., 2013; Tournas and Katsoudas, 2008), fungal contaminants, or aflatoxins (Esmi et al., 2022; Özden and Özden, 2018; Reinholds et al., 2020), raising concerns about ocular infections. Given the delicate nature of the ocular surface, official policies should discourage direct ocular application of chamomile, especially without pharmaceutical-grade sterilization.

### 3.5 Microbial Contamination Screening

Chamomile teas intended for medicinal use (not ophthalmic, but minor skin washes etc.) must undergo rigorous microbial contamination screening to ensure they meet “sterility” (low-germ) standards. This includes testing for bacteria (especially pathogenic), fungi, and endotoxins.

### 3.6 Sterilization Protocols

Approved ocular herbal remedies should follow established sterilization protocols such as sterile filtration, bulk heat sterilization, and aseptic compounding to achieve pharmaceutical-grade purity. For approved ophthalmic solutions, guidelines such as those provided by the *American Academy of Ophthalmology* (AAO), *the American Society of Health-System Pharmacists* (ASHP) and *European Pharmacopoeia* (Ph. Eur. 11.0) emphasize the importance of sterility and quality control. These methods ensure that the products are free from contaminants that could cause infections or other complications.

### 3.7 Quality Control

For container closure integrity testing, the *FDA Guidance Document* provides recommendations for using methods other than sterility testing to confirm container and closure system integrity as part of the stability protocol for sterile products. Additionally, *21 CFR 211.94* outlines specific requirements for medical product containers and closures, including standards for cleanliness, sterilization, and protection against contamination. For sterilization process validation, the FDA’s *Sterilization Process Controls* guidelines emphasize the importance of validating sterilization processes, controlling and monitoring these processes, and ensuring personnel are appropriately qualified. *FDA The Guidance for Industry: Sterile Drug Products Produced by Aseptic Processing* provides detailed recommendations for the sterilization and production of sterile drug products using aseptic processing techniques.

### 3.8 Metallic contamination

Traditional herbal remedies have been linked to heavy metal contamination due to their organic metal chelating characteristics (Hrkić et al., 2021; Kovács-Valasek et al., 2023; Özden and Özden, 2018; Rák and Csutak, 2024a; Shimshoni et al., 2015; Singh et al., 2011). EMA guidelines do not adequately address the specific risks associated with *M. recutita* exposure to the eye. A policy shift is needed to explicitly warn against ocular use in patient information leaflets.

### 3.9 Lack of clinical awareness and public guidance

While clinicians caution against *M. recutita* poultices due to cross-reactivity risks (Andres et al., 2009; Reider et al., 2000), many patients remain unaware of these hazards. The lack of precise epidemiological data on allergic and toxic ocular reactions hampers evidence-based policy making. Greater emphasis on public education and clearer contraindications in monographs and herbal medicine guidelines would be beneficial.

### 3.10 Healthcare provider guidelines

Healthcare provider guidelines: Evidence-based clinical guidelines must be developed to discourage chamomile poultice use for ophthalmic conditions. Calls and information from professional organizations, such as the AAO, regarding practices that pose risks to patients (e.g., chamomile eye poultices) must be disseminated. These topics must be included in continuing education programs to ensure healthcare providers are well-informed and can effectively communicate these risks to patients. Ophthalmologists and other medical specialists must be encouraged to educate patients on the risks associated with self-administered herbal treatments.

### 3.11 Public health education campaigns

Public health education campaigns: Public health education campaigns must be launched through pharmacies, herbal medicine retailers, and public health agencies to inform consumers of the potential allergic and microbial risks. Alternative, scientifically validated treatments for common ocular ailments that patients often attempt to treat with *M. recutita* must be provided.

### 3.12 Further research and surveillance

In some countries, smaller regional surveys have been conducted to assess the use of herbal and other complementary and alternative therapies among ophthalmic patients (AlSalman et al., 2021; Kebede et al., 2021; Wan et al., 2010; Yurdakul, 2024). However, in Hungary and other countries around the world, there has been no comprehensive epidemiological survey, leaving certain risk factors and public health information hidden. Currently, the authors are planning a larger registry on this topic at the University of Pécs. Large-scale epidemiological studies must be conducted to quantify the prevalence of ocular allergic reactions and infections linked to herbal remedies. A reporting system for adverse ocular events associated with herbal medicine use must be established, aiding policy adjustments based on emerging data. International validated questionnaires (e.g., I-CAM-Q) are encouraged to apply (Quandt et al., 2009).

### 3.13 Endangering patients and sanctions

In the European Union, the authorization and supervision of medicines and therapies are regulated by *Directive 2001/83/EC* of *the European Parliament and the Council*, as well as the *EMA*. In Hungary, the authorization and supervision of medicines and therapies are carried out by the *National Public Health Center* (NNK) and the *National Institute of Pharmacy and Nutrition* (OGYÉI) and regulated by *Act CLIV of 1997 on Health*. The directives stipulate that only medicines and therapies that meet safety and efficacy requirements can be used. In the case of unauthorized therapies, sanctions may include product recalls, fines, and criminal liability for responsible individuals. Based on the information gathered in this article, it is necessary that in the future, healthcare professionals be held accountable for endangering a patient’s health with chamomile poultice for eye treatment, especially if complications threatening vision or life arise.

By implementing these recommendations, policymakers and healthcare professionals can reduce the risks associated with chamomile and similar traditional remedies, ultimately improving ocular health outcomes and preventing preventable cases of severe ocular infection and inflammation.

## 4 Discussion

Chamomile poultices generate allergic symptoms that disguise the primary ophthalmic complaint (e.g., blepharitis, hordeolum, bacterial conjunctivitis, etc.), hence discontinuing chamomile poultices is the first line of treatment and patient education. Its therapy is local, and preservative-free artificial tears play an important part. These reduce subjective symptoms, dilute inflammatory substances in tears, and remove discharge from the eye socket. Eyedrops containing antihistamines and mast cell stabilizers are examples of targeted therapy (Módis and Süveges, 2023). Anti-inflammatory therapy should not be initiated until there is extensive involvement of the ocular surface epithelium, as a delay may result in permanent damage. The preservative-free hydrocortisone topical steroid is effective for the treatment of ocular surface inflammatory diseases (dry eye, allergic conjunctivitis, ocular surface inflammation associated with autoimmune diseases) without significantly increasing intraocular pressure or resulting in cataracts (Tóth-Molnár, 2023). Central European pharmacies provide sterile high quality polyherbal preparations containing *M. recutita* extract, including the active ingredient α-bisabolol, such as Iridina Green^®^ (COC Farmaceutici, Italy), Eumill^®^ (COC Farmaceutici, Italy), Gabriel^®^ (Béres Gyógyszergyár, Hungary), and Lacrimal^®^ Natura (Polpharma, Poland) (Rák and Csutak, 2024b) etc. *M. recutita* extract is present in Puralid^®^ Lipogel (Santen Oy, Finland) eyelid cream, which is administered to treat chronic or *Demodex* spp.-induced eyelid inflammation, and Alantel^®^ (Nakafarma, Spain), an eyelid cream developed to treat radiation dermatitis and atopic eyelid inflammation caused by anti-glaucoma eye drops. Oculocin^®^ Propo (Origmed, Lithuania) eye drops are unique in that they contain propolis in addition to *M. recutita* active components, albeit there is limited proof of their effectiveness in ophthalmology (Rák and Csutak, 2024b). These are sterile preparations; thus, the allergenic effect is negligible. In case of known allergies to Asteraceae plants, performing a skin test on a more neutral area (e.g., supine surface of hand or forearm) is recommended. Experiencing minor redness or itching, the product must be discontinued. Regarding many medications containing active medicinal plant(s) are available in Central European pharmacies to treat dry eye and eyelid inflammations, their descriptions are beyond the scope of this review.

In conclusion, the assessment of policies and guidelines related to the ocular application of traditional herbal remedies, particularly *M. recutita* poultices, must consider the risks of allergic reactions, microbial contamination, and chemical damage to the ocular surface. Herbal tea made from *M. recutita* has been used in certain cultures for the treatment of eye inflammations since ancient Greek-Roman-Unani medicine. In our current era, cognitive dissonance has developed between scientific evidence and superstitious-cultural folk beliefs, which this publication aims to begin resolving. Due to its public health importance and the exposure of patients to risks, it does not receive enough emphasis from official authorities. In ophthalmic patient care, we require an integrative approach as well as the expertise of ophthalmologists and pharmacists who understand phytotherapy, because, in addition to individual sensitivity, several drug-herb interactions have been identified (Rák and Csutak, 2024a; 2024b). The authors hope that this article has prompted experts to act against unproven and harmful traditional folk treatments for eye diseases. Maybe 1 day, the chamomile eye poultice might become medical history data for future generations.

## Abbreviations

AAO, American Academy of Ophthalmology; APCs, Antigen-Presenting Cells; ASHP, American Society of Health-System Pharmacists; CALT, Conjunctival-Associated Lymphoid Tissue; COX, Cyclooxygenase; EC, European Commission; EMA, European Medicines Agency; ESCOP, European Scientific Cooperative on Phytotherapy; FDA, Food and Drug Administration; GRAS, Generally Recognized as Safe; I-CAM-Q, International Complementary and Alternative Medicine Questionnaire; IgA, Immunoglobulin A; IgE, Immunoglobulin E; IL-33, Interleukin 33; LDALT, Lacrimal Drainage-Associated Lymphoid Tissue; MMP-9, Matrix etalloproteinase-9; OCT, Optical Coherence Tomography; Ph. Eur., European Pharmacopoeia; TGF-β, Transforming Growth Factor Beta; WHO, World Health Organization.

## References

[B1] Aaqil A. M. Mahboob S. Nafees K. M. (2022). Phytochemical and pharmacological studies and evidence-based indications of babuna (Matricaria chamomilla L.): a review. AYUSHDHARA 8, 3700–3708. 10.47070/ayushdhara.v8i6.857

[B2] Ács M. (1930). About the management of genital fluor. Orv. Hetil. 74, 1327. [A fluorkezelésről].

[B3] Alan Andersen F. (1999). Final report on the safety assessment of bisabolol. Int. J. Toxicol. 18, 33–40. 10.1177/109158189901800305 16835129

[B4] Al-Ghadeer H. Al-Amry M. (2021). Ocular complications resulting from the use of traditional herbal medicine in Central Saudi Arabia: a review. Middle East Afr. J. Ophthalmol. 28, 131–136. 10.4103/MEAJO.MEAJO_120_21 34759672 PMC8547670

[B5] AlSalman S. AlHussaini M. A. Khandekar R. B. Edward D. P. (2021). The proportion of complementary and alternative medicine utilization among Saudi population for eye care: cross-sectional study. Cureus 13, e13109. 10.7759/CUREUS.13109 33728129 PMC7935158

[B6] Andres C. Chen W. C. Ollert M. Mempel M. Darsow U. Ring J. (2009). Anaphylactic reaction to camomile tea. Allergol. Int. 58, 135–136. 10.2332/ALLERGOLINT.C-08-63 19050375

[B7] Atawi R. Ayed A. Batran A. (2024). Traditional eye medicine practice and its determinant factors among ophthalmic patients in the West Bank. J. Public health Res. 13, 22799036241243267. 10.1177/22799036241243267 38577243 PMC10993683

[B8] Balázs E. Nagy F. Váby I. (1962). Congenital upper eyelid inversion. Szemeszet 99, 157–159. 13864268

[B9] Barcsay-Veres A. (2022). The role of bioprotection in the treatment of dry eyes. [Bioprotekció szerepe a szemszárazság kezelésében]. Szemészet - Ophthalmol. Hung. 159.

[B10] Barlay J. (1904). Cysticercus cellulosae in the anterior chamber. [Cysticercus cellulosae az elülső csarnokban]. Szemeszet 41, 98–99.

[B11] Békés D. (1910). Two cases of acute dacryoadenitis. Dacryoadenitis acuta két esete. Szemeszet 47, 117–118.

[B12] de Paiva C. S. St. Leger A. J. Caspi R. R. (2022). Mucosal immunology of the ocular surface. Mucosal Immunol. 15, 1143–1157. 10.1038/S41385-022-00551-6 36002743 PMC9400566

[B13] EMA (2015). European Union herbal monograph on Matricaria recutita L., flos. Eur. Med. Agency.

[B14] Erdei A. Papp N. Mihalik E. (2011). The heritage of our ancestors: ethnobotanical evaluation of settlements in Békés and Székely Land. Szeged, Hungary: University of Szeged. [Elődeink öröksége: békési és székelyföldi települések etnobotanikai értékelése].

[B15] Erdész F. Kozak A. (2008). Medicinal plant sector in Hungary. [A gyógynövényágazat helyzete]. Budapest.

[B16] ESCOP (2020). ESCOP monographs: matricariae flos (Matricaria flower).

[B17] Esmi F. Khoshnamvand Z. Nazari F. Tajkey J. Khosrokhavar R. Mohseni M. (2022). Ochratoxin A in chamomile, black and green tea and human health risk assessment in Iranian population. J. Food Meas. Charact. 16, 5066–5076. 10.1007/s11694-022-01584-y

[B18] Fazekas P. (2010). Telemedicine: healing or guidance? [Gyógyítás vagy tanácsadás a távgyógyászat?]. Inf. Tarsad. 10, 48–52. 10.22503/INFTARS.X.2010.3-4.4

[B19] Florido-Lopez J. F. Gonzalez-Delgado P. De San Pedro B. S. Perez-Miranda C. De Saavedra J. M. A. Marin-Pozo J. F. (1995). Allergy to natural honeys and camomile tea. Int. Arch. Allergy Immunol. 108, 170–174. 10.1159/000237135 7549505

[B20] Fraunfelder F. T. (2008). Herbal medicine and dietary supplement induced ocular side effects. Clin. Ocul. Toxicol. Drug-Induced Ocul. Side Eff., 307–313. 10.1016/B978-1-4160-4673-8.10009-9

[B21] Fraunfelder F. W. (2004). Ocular side effects from herbal medicines and nutritional supplements. Am. J. Ophthalmol. 138, 639–647. 10.1016/j.ajo.2004.04.072 15488795

[B22] García J. A. Rosas J. E. García y Santos C. Streitenberger N. Feijoo M. Dutra F. (2020). Senecio spp. transboundary introduction and expansion affecting cattle in Uruguay: clinico-pathological, epidemiological and genetic survey, and experimental intoxication with Senecio oxyphyllus. Toxicon 173, 68–74. 10.1016/J.TOXICON.2019.11.013 31785285

[B23] Guzelmeric E. Ristivojević P. Vovk I. Milojković-Opsenica D. Yesilada E. (2017). Quality assessment of marketed chamomile tea products by a validated HPTLC method combined with multivariate analysis. J. Pharm. Biomed. Anal. 132, 35–45. 10.1016/J.JPBA.2016.09.030 27693951

[B24] Györfi A. (2016). The relation of healthcare system with magical thinking. [Az egészségügy és a mágikus gondolkodás viszonya]. Orv. Hetil. 157, 2088–2092. 10.1556/650.2016.HO2557 28019115

[B25] Hrkić M. Murtić M. S. Murtić S. (2021). A quality evaluation of chamomile and mint teas commonly consumed in Bosnia and Herzegovina. J. Excipients Food Chem. 12, 41–48.

[B26] Jarić S. Kostić O. Mataruga Z. Pavlović D. Pavlović M. Mitrović M. (2018). Traditional wound-healing plants used in the Balkan region (Southeast Europe). J. Ethnopharmacol. 211, 311–328. 10.1016/j.jep.2017.09.018 28942136

[B27] Jost P. (2022). Plants and weeds - in natural medicine. [Növények és gaz - a természetes gyógykezelésben]. Budapest: Nemzeti Örökség.

[B28] Kebede E. B. Tan J. Iftikhar S. Abu Lebdeh H. S. Duggirala M. K. Ghosh A. K. (2021). Complementary and alternative medicine use by patients from the gulf region seen in the international practice of a tertiary care medical center. Glob. Adv. Heal. Med. 10. 10.1177/21649561211010129 PMC8076768 33996270

[B29] Keller S. E. Stam C. N. Gradl D. R. Chen Z. Larkin E. L. Pickens S. R. (2015). Survival of Salmonella on chamomile, peppermint, and green tea during storage and subsequent survival or Growth following tea brewing. J. Food Prot. 78, 661–667. 10.4315/0362-028X.JFP-14-508 25836389

[B30] Kerckhoff A. (2020). Treben, Maria (1907–1991). Wichtige Frauen Naturheilkd, 163–177. 10.1007/978-3-662-60459-5_25

[B31] Kovács-Valasek A. Rák T. Pöstyéni E. Csutak A. Gábriel R. (2023). Three major causes of metabolic retinal degenerations and three ways to avoid them. Int. J. Mol. Sci. 24, 8728. 10.3390/IJMS24108728 37240082 PMC10218427

[B32] Leitner V. (1903). Literature review. Szemeszet 40, 15.

[B33] Leitner V. (1905). About the therapy of neonatal blenorrhoea. [Blennorrhoea neonatorum therápiájáról]. Szemeszet 42, 344–353.

[B34] Liu Y. Wu F. Zhu Y. Chen Y. Murray K. Lu Z. (2020). Survival of toxigenic *Escherichia coli* on chamomile, peppermint, green, black, ginger, and cinnamon teas during storage and brewing. J. Food Saf. 40, e12831. 10.1111/JFS.12831

[B35] Maji A. K. Banerji P. (2015). Chelidonium majus L. (greater celandine) - a review on its phytochemical and therapeutic perspectives. Int. J. Herb. Med. 3, 10–27. 10.22271/FLORA.2015.V3.I1.03

[B36] Makay B. Kiss J. (1988). Folk healings in szatmár. [Népi gyógyítások szatmárban]. 1st ed. Budapest: Népszava Kiadó.

[B37] McKay D. L. Blumberg J. B. (2006). A review of the bioactivity and potential health benefits of chamomile tea (Matricaria recutita L.). Phyther. Res. 20, 519–530. 10.1002/PTR.1900 16628544

[B38] Mihyaoui A.El Esteves Da Silva J. C. G. Charfi S. Castillo M. E. C. Lamarti A. Arnao M. B. (2022). Chamomile (Matricaria chamomilla L.): a review of ethnomedicinal use, phytochemistry and pharmacological uses. Life 12, 479. 10.3390/LIFE12040479 35454969 PMC9032859

[B39] Módis L. Süveges I. (2023). A szemfelszín allergiás és immunpatológiai betegségei. Orv. Hetil. 164, 1686–1692. 10.1556/650.2023.32910 37898906

[B40] Navarro-Triviño F. J. Ayén-Rodríguez Á. Ruiz-Villaverde R. (2022). Contact urticaria caused by chamomile in a wet wipe. Contact Dermat. 86, 548–549. 10.1111/COD.14067 35122256

[B41] OGYÉI (2023). Plants not recommended for use in food and dietary supplements by the Scientific Advisory Board of the NPFHI. Az OGYÉI Tudományos Tanácsadó Testülete által élelmiszerekben, étrend-kiegészítőkben alk. nem. javasolt növények. Available online at: https://ogyei.gov.hu/dynamic/alkalmazasra_nem__javasolt_novenyek_20220609.pdf.

[B42] Özden H. Özden S. (2018). Levels of heavy metals and ochratoxin A in medicinal plants commercialized in Turkey. Turk. J. Pharm. Sci. 15, 376–381. 10.4274/TJPS.74936 32454685 PMC7227822

[B43] Parveen A. Perveen S. Naz F. Ahmad M. Khalid M. (2023). Chamomile. Aromat. Crop., 1009–1040. 10.1007/978-3-031-35403-8_39

[B44] Paulsen E. Christensen L. P. Andersen K. E. (2008). Cosmetics and herbal remedies with Compositae plant extracts – are they tolerated by Compositae-allergic patients? Contact Dermat. 58, 15–23. 10.1111/J.1600-0536.2007.01250.X 18154553

[B45] Quandt S. A. Verhoef M. J. Arcury T. A. Lewith G. T. Steinsbekk A. Kristoffersen A. E. (2009). Development of an international questionnaire to measure use of complementary and alternative medicine (I-CAM-Q). J. Altern. Complement. Med. 15, 331–339. 10.1089/ACM.2008.0521 19388855 PMC3189003

[B46] Rák T. Csutak A. (2024a). Complementary practices in pharmacy and their relation to glaucoma—classification, definitions, and limitations. Sci. Pharm. 92, 16. 10.3390/SCIPHARM92010016

[B47] Rák T. Csutak A. (2024b). Exploring novel pharmacological trends: natural compounds in dry eye disease management. Acta Pharm. 74, 383–404. 10.2478/ACPH-2024-0028 39279530

[B48] Ramazani E. Akaberi M. Emami S. A. Tayarani-Najaran Z. (2022). Pharmacological and biological effects of alpha-bisabolol: an updated review of the molecular mechanisms. Life Sci. 304, 120728. 10.1016/J.LFS.2022.120728 35753438

[B49] Reider N. Sepp N. Fritsch P. Weinlich G. Jensen-Jarolim E. (2000). Anaphylaxis to camomile: clinical features and allergen cross-reactivity. Clin. Exp. Allergy 30, 1436–1443. 10.1046/J.1365-2222.2000.00902.X 10998021

[B50] Reinholds I. Bogdanova E. Pugajeva I. Alksne L. Stalberga D. Valcina O. (2020). Determination of fungi and multi-class mycotoxins in camelia sinensis and herbal teas and dietary exposure assessment. Toxins (Basel) 12, 555. 10.3390/TOXINS12090555 32872457 PMC7551389

[B51] Sah A. Naseef P. P. Kuruniyan M. S. Jain G. K. Zakir F. Aggarwal G. (2022). A comprehensive study of therapeutic applications of chamomile. Pharmaceuticals 15, 1284. 10.3390/PH15101284 36297396 PMC9611340

[B52] Santaella R. M. Fraunfelder F. W. (2007). Ocular adverse effects associated with systemic medications: recognition and management. Drugs 67, 75–93. 10.2165/00003495-200767010-00006 17209665

[B53] Shimshoni J. A. Duebecke A. Mulder P. P. J. Cuneah O. Barel S. (2015). Pyrrolizidine and tropane alkaloids in teas and the herbal teas peppermint, rooibos and chamomile in the Israeli market. Food Addit. Contam. Part A 32, 2058–2067. 10.1080/19440049.2015.1087651 26365752

[B54] Silva D. I. D. Araujo R. O. Colaço W. Freire L. (2013). Isolation of resistant bacteria from commercial samples of camomile (Matricaria recutita).

[B55] Singh O. Khanam Z. Misra N. Srivastava M. K. (2011). Chamomile (Matricaria chamomilla L.): an overview. Rev. 5, 82–95. 10.4103/0973-7847.79103 PMC3210003 22096322

[B56] Srivastava J. K. Shankar E. Gupta S. (2010). Chamomile: a herbal medicine of the past with bright future. Mol. Med. Rep. 3, 895–901. 10.3892/MMR.2010.377 21132119 PMC2995283

[B57] Subiza J. Subiza J. L. Hinojosa M. Garcia R. Jerez M. Valdivieso R. (1989). Anaphylactic reaction after the ingestion of chamomile tea: a study of cross-reactivity with other composite pollens. J. Allergy Clin. Immunol. 84, 353–358. 10.1016/0091-6749(89)90420-X 2674263

[B58] Süveges I. (2020). “Ophthalmology. Szemészet,” in Medicina kiadó zrt. 5th ed. Budapest.

[B59] Sváb J. (1979). New aspects of cultivating chamomile. Herba Pol. 25, 35–39.

[B60] Szabó L. G. (2016). Medicinal plant of the year: chamomile - the history of Hungarian chamomile research. [Az év gyógynövénye: orvosi székfű – a magyar kamillakutatás múltja]. Farmakognóziai Hírek 11, 3–5.

[B61] Torday F. (1872). Keratitis pustulosa. Szemeszet 9, 13–14.

[B62] Tóth-Molnár E. (2023). Új fejezet a szemfelszín gyulladásos betegségeinek kezelésében: soft típusú topikális szteroid a gyulladáscsökkentő terápiás algoritmusban. Szemeszet 160, 13–18. 10.55342/SZEMHUNGARICA.2023.160.1.13

[B63] Tournas V. H. Katsoudas E. J. (2008). Microbiological quality of various medicinal herbal teas and coffee substitutes. Microbiol. Insights 1. 10.4137/MBI.S943

[B64] Travassos A. R. Goossens A. (2012). Potential allergens in moisturizing creams. Syndr. Art. Sci. Moisturizers, 367–378. 10.1007/978-3-642-27606-4_24

[B65] Treben M. (2023). Health through God’s pharmacy. Budapest: Kötet Kiadó.

[B66] Ujsághy P. (1943). Therapy of toxicosis in infancy. Orv. Hetil. 87, 7–8.

[B67] Vámos G. (2024). “They blew sugar on it to make it hurt less.” Details of the use of sugar in folk eye treatment in the 18th-20th centuries. [“Fújtak rá cukrot, hogy ne fájjon annyira.” Adatok a cukor népi szemgyógyításban betöltött szerepéhez a 18-20. században]. Kaleidosc. Hist. 14, 320–333. 10.17107/KH.2024.28.17

[B68] Vandenplas O. Pirson F. D’Alpaos V. Borght T. V. Thimpont J. Pilette C. (2008). Occupational asthma caused by chamomile. Allergy 63, 1090–1092. 10.1111/J.1398-9995.2008.01752.X 18691315

[B69] Vidor Z. (1879). The mercury remedy for eye diseases. A higany-gyógymód szembetegségeknél. Orv. Hetil. 23, 72–76.

[B70] Wan M. J. Daniel S. Kassam F. Mutti G. Butty Z. Kasner O. (2010). Survey of complementary and alternative medicine use in glaucoma patients. J. Glaucoma 1, 79–82. 10.1097/IJG.0b013e3182027c0c 21173701

[B71] Weinstein P. (1957). Book review. Szemeszet 94, 187.

[B72] Wesolowska A. Grzeszczuk M. Kulpa D. (2015). Propagation method and distillation apparatus type affect essential oil from different parts of Matricaria recutita L. Plants. J. Essent. Oil Bear. Plants 18, 179–194. 10.1080/0972060X.2014.895210

[B73] Wilkinson S. M. Hausen B. M. Beck M. H. (1995). Allergic contact dermatitis from plant extracts in a cosmetic. Contact Dermat. 33, 58–59. 10.1111/J.1600-0536.1995.TB00457.X 7493472

[B74] World Health Organization (2002). Traditional medicine: growing needs and potential. Geneva.

[B75] World Health Organization (2019). WHO global report on traditional and complementary medicine. Geneva, Switzerland: World Health Organization.

[B76] Yamoah F. O’Caoimh C. Donnelly C. Sawaya S. (2014). The journey from subsistence to commercial viability: the case of meru herbs, Kenya. Nternational food agribus. Manag. Rev. 17, 139–144.

[B77] Yurdakul B. (2024). Alternative therapies for eye inflammation: patient preferences and patterns. Int. J. Tradit. Complement. Med. Res. 5, 47–53. 10.53811/IJTCMR.1432912

[B78] Zörgő S. Purebl G. Zana Á. (2016). Factors determining selection of treatment options oriented towards complementary and alternative medicine. [A komplementer és alternatív medicina felé orientálódó terápiaválasztást meghatározó tényezők]. Orv. Hetil. 157, 584–592. 10.1556/650.2016.30402 27039997

