# Supplementary figures and images for: Reevaluating the safety of chamomile poultices in ophthalmic care
Source: Front Pharmacol. 2025 May 12;16:1580586. doi: 10.3389/fphar.2025.1580586 (PMC12104180; doi:10.3389/fphar.2025.1580586)

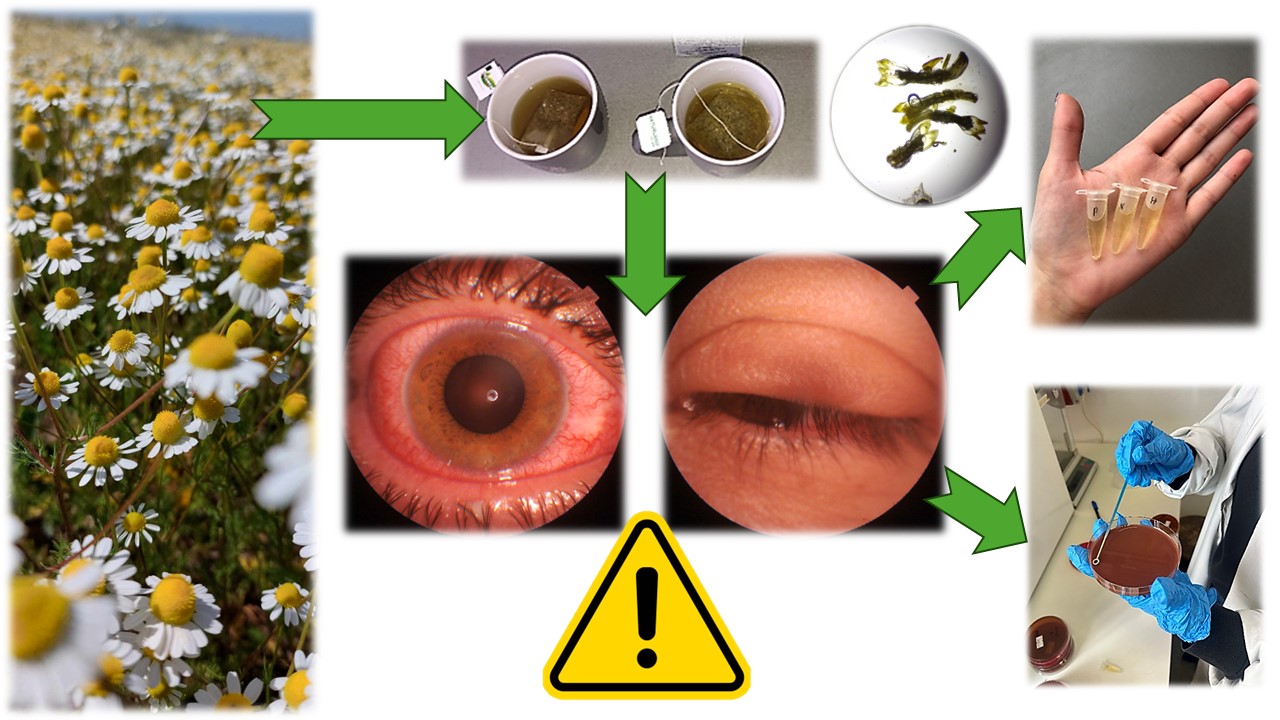

Supplement: Supplementary file 1 [file Image1.jpeg]
